# Comparative Study of *Berberis vulgaris* Fruit Extract and Berberine Chloride Effects on Acetic Acid-Induced Colitis in Rats

**Published:** 2011

**Authors:** Mohsen Minaiyan, Alireza Ghannadi, Parvin Mahzouni, Elham Jaffari-Shirazi

**Affiliations:** a *Department of Pharmacology, School of Pharmacy and Pharmaceutical Sciences, Isfahan University of Medical Sciences, Isfahan, Iran.*; b * Department of Pharmacognosy, School of Pharmacy and Pharmaceutical Sciences, Isfahan University of Medical Sciences, Isfahan, Iran.*; c *Department of Clinical Pathology, School of Medicine, Isfahan University of Medical Sciences, Isfahan, Iran.*; d * Isfahan Pharmaceutical Sciences Research Center, Isfahan, Iran.*

**Keywords:** *Berberis vulgaris*, Berberine, Barberry fruit extract, Colitis, Rats

## Abstract

Antioxidant and immunomodulatory effects of anthocyanins are abundant in berberry fruits suggesting that they may have beneficial effects on inflammatory bowel diseases (IBD). The present study was carried out to investigate the anti-colitic effect of *Berberis vulgaris *fruit extract (BFE) compared to berberine chloride (BEC) and corticosteroids using an animal model of acetic acid induced experimental colitis. BFE with three different doses (375, 750, and 1500 mg/Kg) was administered orally or rectally prior to ulcer induction. BEC (10 mg/Kg), prednisolone (5 mg/Kg), hydrocortisone acetate enema (20 mg/Kg) and normal saline (5 mL/Kg) were considered as respective controls. The tissue was assessed macroscopically for damage scores, area, index and weight/length ratio. They were also examined histopathologically for inflammation extent and severity, crypt damage, invasion involvement and total colitis index.

Results indicated that greater doses of oral BFE (750, 1500 mg/Kg) as well as BEC (10 mg/Kg) were effective to protect against colonic damage. By rectal pretreatment, the extract was only effective to diminish the ulcer index and the efficacy was not significant for mucosal inflammation parameters. In conclusion BFE, which is nearly devoid of berberine, was effective to protect against colitis and this might be attributed to its anthocyanin constituents.

## Introduction

Inflammatory bowel disease (IBD) is characterized by chronic intestinal inflammation and can be found in two forms: Crohn’s disease and ulcerative colitis. Although immunologic mechanisms have been postulated as an important participant in these diseases, their ethiology and pathophysiology are still unknown. Sulfasalazine, mesalamine and 5-ASA derivatives, glucocorticoides and immuno-suppressives are among the current medications for which limited efficacy and various side effects are common (1). Because of the lack of specific and curative treatments with limited toxicity, there is a growing need to develop safe and effective therapeutic approaches to IBD (2).

*Berberis vulgaris *is a shrub in the family Berberidaceae, native to central and southern Europe, northwest Africa and western Asia. The fruit is an oblong red berry 7-10 mm long and 3-5 mm broad, ripening in late summer or autumn; they are edible but very sour, and rich in vitamin C. In southwestern Asia, especially in Iran, where they are called *zereshk*, the berries are commonly used for cooking as well as for making jam so the production of fresh edible seedless barberries fruit reaches to about 22000 tons annually (3, 4).

*Berberis vulgaris *as well as other berberine (BE) containing plants (5) are used medicinally in virtually all-traditional medical systems, and have a history of usage in Ayurvedic, Iranian and Chinese medicine dating back at least 3,000 years (6). Phytochemical analysis of root or stem bark extract of *B. vulgaris *demonstrated the presence of protoberberines and bisbenzyl-isoquinoline alkaloids (berbamine, tetrandrine and chondocurine) for which anti-inflammatory and immuno-suppressive activities have also been well established (7). Berberine, an isoquinoline alkaloid and the major ingredient of this plant, has been used for treating diarrhea and gasterointestinal disorders for a long time (8, 9). It has multiple pharmacological effects including; antimicrobial activity against 54 microorganisms (10-12), inhibition of intestinal ion secretion and smooth muscle contraction, inhibition of ventricular tachyarrhythmias, reduction of inflammation, stimulation of bile secretion and bilirubin discharge (13). Among berberine multiple pharmacological actions, anti-inflammatory activity has been extensively studied (14). Antipyretic activity of berberine sulfate has also been shown by Sabir and Bhide (1971) using a model of experimentally induced fever in rats (13). This effect has been found to be approximately three times greater than sodium salicylate. Anti-colitic property is another pharmacological effect has been demonstrated for berberine by Zhou and Mineshita (15).

On the other hand, barberry fruit, the flowers, fruit pulp and the seeds have little or no significant amount of alkaloids however; they contain a great amount of phenol compounds, gum, pectin, oleoresins and organic acids (16). The barberry phenol compounds include anthocyanins and carotenoid pigments (4, 17). Several pharmacological effects such as antioxidant and cytoprotective (18), inhibitory effects on capillary permeability (19) and epidermal growth factor (20), anticholinergic and antihistaminergic (17), have been demonstrated for anthocyanins and berberry fruit extract (BFE). Therefore Barberry fruit might be a very promising alternative medicine or functional food for IBD therapy or prevention probably by altering the mechanisms might involve in disease pathology. The aim of this study was to investigate the protective effect of BFE (orally and rectally) on acute colitis induced by acetic acid in comparison to berberine chloride (BEC) and glucocorticoids.

## Experimental

*Animals*

Male Wistar rats (Razi Institute, Tehran, Iran) weighting 200-250 g were used in this study. The animals were accommodated separately in wire-bottomed cages under a uniform condition of light/dark cycle (12 h/12 h), temperature (20 ± 4°C) and humidity (50-70%) with normal rat chow and tap water *ad libitum*. All rats were fasted for 36 hrs prior to the experimental procedure. All animal procedures were carried out using protocols approved by local ethical committee of Isfahan University of Medical Sciences. 

*Plant material and preparation of extract*

Barberry fresh fruits (*Berberis vulgaris *var. *asperma*) were prepared from Ghaenaat (Southern Khorasan province, Iran) and authenticated by Pharmacognosy Department of Mashhad Pharmacy School. For preparation of hydroalcoholic extract, dried and finely powdered fruit (1000 g) was soaked by adequate volume of ethanol : water (70 : 30) and the extraction was undertaken for 48 h to obtain full extract using percolation method. The extract was then shuddered, filtered and evaporated in a rotary evaporator under reduced pressure until a semisolid and jam nature extract yielded 37.7% (w/w) (21, 22).

*Chemicals*

Prednisolone powder and hydrocortisone acetate enema were procured from Iran Hormone Pharmaceutical Co. (Tehran, Iran) and Valeant Pharmaceutical Co. (Saint-Laurent, Canada) respectively. Berberine chloride was purchased from Sigma Co. (Sigma, UK). All organic solvents were of analytical grade and Merck brand (Merck, Germany).

*Grouping*

1, 2: Sham groups; Normal saline (5 mL/Kg) was administered orally (p.o.) or rectally (i.r.) prior to intra-colonic instillation of normal saline (as vehicle).

3, 4: Control groups; Normal saline (5 mL/Kg) was administered p.o. or i.r., prior to colitis induction.

5, 6, 7, 8, 9: Extract groups; BFE with doses of 375, 750, and 1500 mg/Kg were administered p.o. for 5 constitutive days and 2 h prior to colitis induction. Two other groups were taken 750 and 1500 mg/kg BFE, as enema, 15 and 2 h prior to colitis induction.

10, 11, 12: Reference groups; prednisolone (5 mg/Kg, p.o.), berberine chloride (10 mg/Kg p.o. and i.r.), and hydrocortisone acetate enema (20 mg/Kg) were administered to other three groups according to respective extract groups. 

*Acute colitis induction*

Colitis was induced in rats by intra-colonic instillation of 2 mL acetic acid 4%. Under light ether anesthesia, a soft pediatric catheter (2 mm inner diameter and 8 cm in length) was introduced into the anus for 8 cm and acetic acid was carefully instilled. Before taking the catheter out, 2 mL air was applied in order to spread the acid completely within the colon (23). 

*Assessment of the colonic damage*

Rats were sacrificed using over-dose ether anesthesia, 24 h after the colitis induction. The abdomen was opened and the colon was exposed. The distal 8 cm of the colon, 2 cm proximal to the anus, was excised and opened by longitudinal incision. After washing the mucosal surface with normal saline, wet weight was measured for each tissue and mucosal injury was assessed macroscopically using the grading scale of Morris *et al*. (24). The scores were: 0 = no; 1 = mucosal erythema only; 2 = mild mucosal edema, slight bleeding or slight erosion; 3 = moderate edema, bleeding ulcers or erosions; 4 = sever ulceration, erosions, edema and tissue necrosis. The ulcer area was measured using 3M^®^ scaled surgical transpore tape, which was fixed on a light and transparent sheet. Each cell on the tape was 1 mm^2^ in area and the number of cells was counted using a magnifier (×4) (Scher, USA) and the ulcer area was determined for each colon. The ulcer index was the later parameter, measured by the addition of the ulcer score and the ulcer area for each tissue specimen (25, 26).

Additional samples were preserved in 10% formalin for histological examination. Formalin-fixed colonic samples were embedded in paraffin and sections were stained with H&E. Inflammation and crypt damage were assessed on H&E-stained, coded the sections using a modification of a validated scoring scheme described by Cooper *et al. *(27) and Dieleman *et al. *(28). Total colitis score is the sum of the 3 subscores (inflammation severity, inflammation extent, and crypt damage). The histologic evaluation and scoring was performed using a Zeiss^®^ microscope equipped with a Sony® color video camera for digital imaging. 

*Statistical analysis*

Results were expressed as the Mean ± SD. Differences among groups were examined using one-way ANOVA with Tukey HSD as post-hoc test. Non-parametric data were analyzed by Mann-Whitney U test. All statistical analysis were performed by SPSS 11 software. Significance was assumed to occur at p < 0.05. 

## Results and Discussion

Using intra-colonic instillation of acetic acid, extensive macroscopic damage of the rat colon was observed compared to sham (normal) groups (Table 1). 

**Table 1:** Effects of *Berberis vulgaris *fruit extract (BFE 375, 750, and 1500 mg/Kg) and berberine chloride (BEC, 10 mg/Kg) on the macroscopic parameters of colitis induced by acetic acid in rats. Prednisolone (Pred., 5 mg/Kg) and hydrocortisone acetate (Hydroc., 20 mg/Kg) were used as reference drugs. The results were expressed as mean ± SD, (n = 6).

**Groups**	**Route**	**Score (0-4)**	**Area (Cm** ^2^ **)**	**w/L**
**Sham**	^a^p.o.	0.0 ± 0.0	0.0 ± 0.0	65.0 ± 3.84***
Control	p.o.	3.66 ± 0.51	5.50 ± 0.87	150.0 ±9.38
BFE375	p.o.	3.16 ± 0.75	4.8 ± 1.09	112.0 ± 7.4**
BFE750	p.o.	2.66 ± 0.75**	4.7 ±1.12	91.3 ± 3.26***
BFE1500	p.o.	1.66 ± 0.81**	3.16 ± 0.89***	86.2 ± 5.74***
Pred.	p.o.	1.33 ± 0.51**	2.16 ± 0.95***	85.0 ± 8.46***
BEC	p.o.	1.50 ± 0.54**	2.66 ± 0.34***	80.0 ± 8.98***
Sham	^b^i.r	0.0 ± 0.0	0.0 ± 0.0	67.8 ± 4.4***
Control	i.r	3.50 ± 0.54	4.98 ± 1.01	140.0 ± 8.98
BFE750	i.r	2.50 ± 0.61*	3.16 ± 0.69*	110.0 ± 8.32**
BFE1500	i.r	2.33 ± 0.66*	3.36 ± 0.45*	107.0 ± 7.80**
Hydroc.	i.r	1.33 ± 0.51**	2.66 ± 0.66***	82.0 ± 6.41***
**BEC**	i.r	1.66 ± 0.51**	2.66 ± 0.68***	60.0 ± 5.79***

^a^orally, ^b^rectally, *p < 0.05, ** p < 0.01, *** p < 0.001 denote significant difference versus control groups

**Figure 1:**
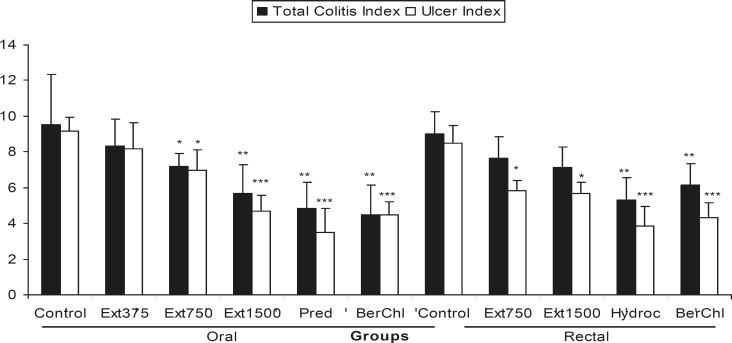
Effect of *Berberis vulgaris *fruit extract (BFE375, 750, 1500 mg/Kg) and berberine chloride (BerChl, 10 mg/Kg) on total colitis index and ulcer index of colon tissue damage induced by acetic acid in rats. Prednisolone (Pred, 5 mg/Kg) and hydrocortisone acetate (Hydroc, 20 mg/Kg) were used as reference agents. The results were expressed as mean ± SD, (n = 6), *p < 0.05, ** p < 0.01, *** p < 0.001 denote significant difference versus control groups

The colonic mucosa both at proximal and distal regions and rectum appeared hemorrhagic and ulcerated. When the macroscopic scores and ulcer index of control groups were compared to those of pretreatment extract groups, it was evident that two greater doses of oral BFE (750, 1500 mg/Kg) were effective to reduce the damage scores and the effect of BFE with the largest dose (1500 mg/Kg) was similar to reference drugs; BEC and prednisolone (Table 1 and Figure 1). Two doses of BEF administered as enema (750, 1500 mg/Kg) were also effective to diminish the damage score, ulcer area, and ulcer index macroscopically however, their efficacy was lower than reference berberine alkaloid and hydrocortisone acetate enema (Table 1 and Figure 1). All of the treatments were effective to decrease colon wet weight / length ratio compared to respected controls (p < 0.001). Regarding to this latter parameter, there was no significant difference between pretreatments and sham-operated groups. 

**Figure 2:**
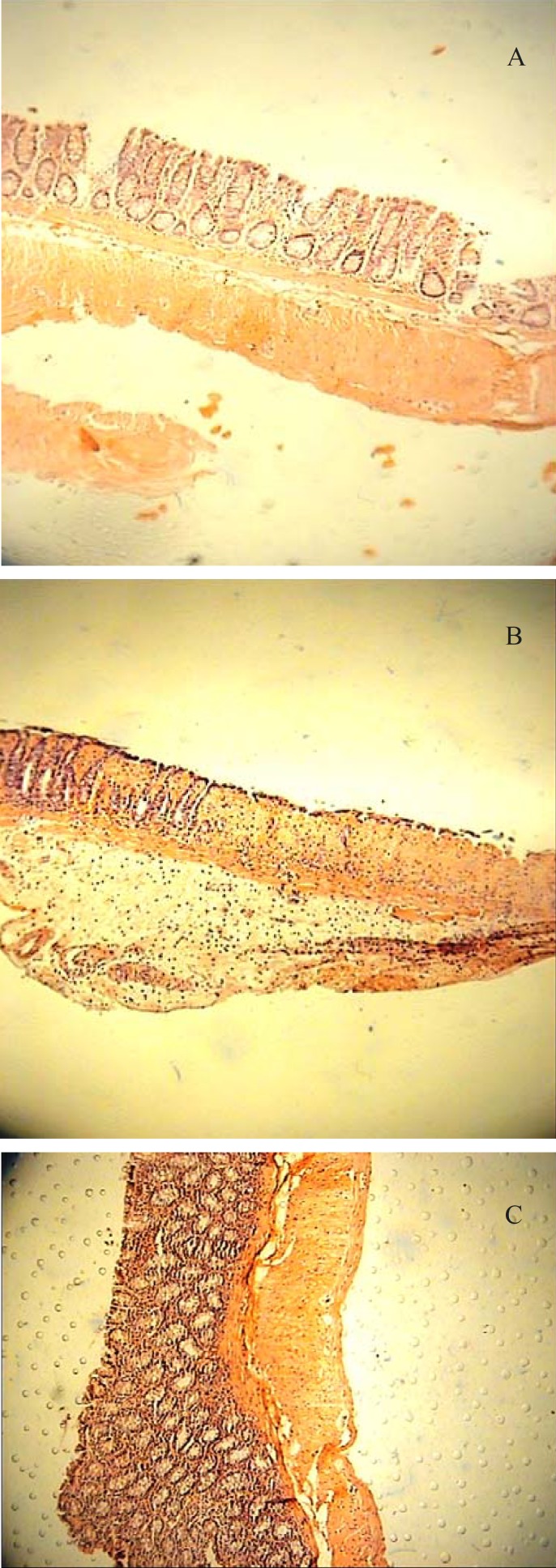
**(**A) Colitis induced by acetic acid in control rats. Erosion of surface epithelium, destruction of crypts and transmural hyperemia, edema and acute inflammation is evident (H&E section, low power). (B) Acetic acid-induced colitis treated with BFE (1500 mg/Kg); the mononuclear cell infiltrate of the lamina propria is much diminished but there is slight edema separating the crypts. All signs of acute inflammation have disappeared (H&E section, low power). (C) Acetic acid colitis treated with prednisolone (5 mg/Kg); the inflammation and crypt injury have been subsided significantly (H&E section, low power).

The colonic damage as determined pathologically, paralleled to that of macroscopically visible injures (Figure 1). Wide areas of epidermal and goblet cell loss and marked inflammatory cell infiltration, ulcers, and crypt damage were observed in the colon specimens of acetic acid treated rats (Figure 2.A). Our findings suggested that two greater doses of BFE (750, 1500 mg/Kg) administered orally were the only extract treatments could prevent against tissue damage histopathologically (Table 2, Figure 1 and Figure 2.B). Prednisolone, BEC and hydrocortisone acetate enema were also effective to reduce pathological damage parameters after p.o. or i.r. pretreatments (Table 2, Figure 1 and Figure 2.C). BFE with the dose of 375 mg/Kg was not effective for any damage score parameters (Table 2 and Figure 1). 

**Table 2:** Effects of *Berberis vulgaris *fruit extract (BFE375, 750, and 1500 mg/Kg) and berberine chloride (BEC, 10 mg/Kg) on the histopathologic parameters of colitis induced by acetic acid in rats. Prednisolone (Pred., 5 mg/Kg) and hydrocortisone acetate (Hydroc., 20 mg/Kg) were used as reference drugs. The results were expressed as mean ± SD, (n = 6).

**Groups**	**Route**	**Inflammation severity**	**Inflammation extent**	**Crypt damage**	**Involvement score**
Sham	^a^p.o.	0.0 ± 0.0	0.0 ± 0.0	0.0 ± 0.0	0.0 ± 0.0
Control	p.o.	2.83 ± 0.40	2.83 ± 0.40	3.83 ±0.40	3.83 ± 0.40
BFE375	p.o.	2.66 ± 0.51	2.50 ± 0.51	3.16 ± 0.75	3.16 ± 0.75
BFE750	p.o.	2.50 ± 0.54	2.33 ± 0.51	2.33 ± 0.51**	2.66 ± 0.51**
BFE1500	p.o.	1.83 ± 0.75*	1.83 ± 0.75*	2.00 ± 0.63**	1.83 ± 0.75**
Pred.	p.o.	1.50 ± 0.54**	1.50 ± 0.54**	1.83 ± 0.75**	1.66 ± 0.51**
BEC	p.o.	1.66 ± 0.81*	1.66 ± 0.51**	1.16 ± 0.75**	1.33 ± 0.81**
Sham	^b^i.r	0.0 ± 0.0	0.0 ± 0.0	0.0 ± 0.0	0.0 ± 0.0
Control	i.r	2.66 ± 0.51	2.66 ± 0.51	3.66 ± 0.51	3.66 ± 0.51
BFE750	i.r	2.33 ± 0.51	2.33 ± o.51	3.00 ± 0.63	2.16 ± 0.40
BFE1500	i.r	2.16 ± 0.66	1.83 ± 0.83	3.16 ± 0.40	2.16 ± 0.3*
Hydroc.	i.r	1.33 ± 0.51**	1.66 ± 0.51*	2.33 ± 0.51**	1.33 ± 0.51**
BEC	i.r	1.66 ± 0.51*	1.83 ± 0.40*	2.66 ± 0.81*	1.66 ± 0.51**

^a^orally, ^b^rectally, *p < 0.05, ** p < 0.01, *** p < 0.001 denote significant difference versus control groups

The acetic acid induced colitis is a rapid, simple, and reproducible model, sharing many characteristics with human colitis (29). Our results confirmed the suitability of method since the ulcerative colitis was induced invariably and prominently in all of the experimental rats. Oral prednisolone and hydrocortisone acetate enema were both effective as reference treatments to protect against experimental acute colitis and the efficacy was independent of route of administration. Glucocorticoids are among the standard drugs for acute colitis treatment and are used extensively in rat models of colitis (30). Their beneficial effects are attributed to their interaction with several inflammatory mediator’s action and/or biosynthesis such as PGs, LTs, and TNF-α (1). Khawashima *et al. *(31) have shown that ameliorating properties of Hange-shashin-to (HST) a traditional herbal formula in Japanese ethnomedicine contained berberine alkaloid as well, which could be attributed to the elevated blood levels of corticosterone on rat experimental colitis. These findings support the suggestion that the protective effects of BFE may be mediated at least partially through a mechanism that resembles glucocorticoides and/or raising blood corticosterone levels. BEC was another reference drug, natural in origin, used to ascertain which active ingredients are probably involved in protective activity of barberry plant and to delineate that further mechanisms might involve in anti-colitic properties of candidate plant. Zhou and Mineshita (15) have shown that berberine chloride itself had beneficial effects on TNBS-induced colitis in rats *in-vitro *and *in-vivo *mainly by inhibiting IL-8 production. Khawashima *et al. *(31) however, failed to show anti-colitic effects of berberine alone probably since they used smaller doses (3.75 and 6.5 mg/Kg) of this alkaloid.

Ivanovska and Philipov (32) on the other hands, have suggested that berberine, as the main constituents of barberry roots and bark extract, had inhibitory effects on carrageenan- induced rat paw edema, acetic acid-induce vascular permeability in rat peritoneal cavity and BCG- induced arthritis. In this later study, berberine beneficial effects were attributed to inhibition of pro-inflammatory mediators over-production including cytokines, nitric oxide and PGE_2_ as well as neutrophil infiltration at sites of inflammation. 

Our findings indicated that the oral BFE with the greatest dose was effective to reduce both the macroscopic and histopathologic damage parameters in such manner was similar to reference drugs. It is assumed that the efficacy was dose-dependent as the middle dose of oral BFE was less effective to diminish histological and macroscopic colitis indices and the lowest dose of BFE was not effective to decrease any parameters of macroscopic or histopathologic assessments.

We also found that the BFE efficacy might be attributed to the route of pretreatment. As it is shown, two greater doses of BFE, which administered orally, were effective to reduce total colitis index however, the same doses used as enema were ineffective. Moreover, oral pretreatment was carried out for 5 days while the rectal administration was made two times a day. This may cause difference in systemic availability and suggesting a higher efficacy for cumulative and delayed protective mechanisms (33). On the other hand, diarrhea was evident in rats with experimental colitis and this resulted in colonic discharge promotion, which could reduce the residence time for active ingredients especially after intra-rectal administration.

It has been shown that BFE as well as the flowers, fruit pulps and seeds, is devoid of significant amount of berberine alkaloid so, BFE efficacy might be attributed to other active components such as anthocyanins to diminish the damage scores (16). Antihistaminic and anticholinergic activities of BFE in the guinea pig ileum have been shown by Shamsa *et al. *(17). More pharmacologic effects such as antioxidant and cytoprotective activity have also been proposed for BFE and anthocyanins (18), which at least some of them may contribute in our findings.

Taken together, our findings indicate that not only BEC as the representative barberry alkaloid but also BFE which is nearly devoid of berberine is effective to reduce tissue damage parameters in the rat model of experimentally induced colitis. This effect is dose-dependent and more evident when the treatments were taken by oral route and for a chronic period and may be attributed to the anthocyanin constituents. This is supportive for barberry fruit and BEC to be used in acute and chronic inflammatory bowel disease in preclinical studies. It is obvious that further studies are required to clarify its mechanism of beneficial effects and warrant their efficacy in clinical setting.
